# Anti-Tumoral Effects of a (1*H*-Pyrrol-1-yl)Methyl-1*H*-Benzoimidazole Carbamate Ester Derivative on Head and Neck Squamous Carcinoma Cell Lines

**DOI:** 10.3390/ph14060564

**Published:** 2021-06-12

**Authors:** Alice Nicolai, Valentina Noemi Madia, Antonella Messore, Daniela De Vita, Alessandro De Leo, Davide Ialongo, Valeria Tudino, Elisabetta Tortorella, Luigi Scipione, Samanta Taurone, Tiziano Pergolizzi, Marco Artico, Roberto Di Santo, Roberta Costi, Susanna Scarpa

**Affiliations:** 1 Department of Experimental Medicine, “Sapienza” University of Rome, Viale Regina Elena 324, 00161 Rome, Italy; alice.nicolai@uniroma1.it (A.N.); elisabetta.tortorella@uniroma1.it (E.T.); susanna.scarpa@uniroma1.it (S.S.); 2 Department of Sensory Organs, “Sapienza” University of Rome, Viale del Policlinico 155, 00161 Rome, Italy; samanta.taurone@uniroma1.it (S.T.); tiziano.pergolizzi@gmail.com (T.P.); marco.artico@uniroma1.it (M.A.); 3 Dipartimento di Chimica e Tecnologie del Farmaco, Istituto Pasteur-Fondazione Cenci Bolognetti, “Sapienza” Università di Roma, P.le Aldo Moro 5, 00185 Rome, Italy; valentinanoemi.madia@gmail.com (V.N.M.); alessandro.deleo@uniroma1.it (A.D.L.); davide.ialongo@uniroma1.it (D.I.); valeria.tudino@uniroma1.it (V.T.); luigi.scipione@uniroma1.it (L.S.); roberto.disanto@uniroma1.it (R.D.S.); roberta.costi@uniroma1.it (R.C.); 4 Department of Environmental Biology, “Sapienza” University of Rome, p.le Aldo Moro 5, 00185 Rome, Italy; daniela.devita@uniroma1.it

**Keywords:** pyrroles, benzimidazoles, nocodazole, head and neck squamous cell carcinoma, apoptosis, epithelial-mesenchymal transition, tubulin

## Abstract

Nocodazole is an antineoplastic agent that exerts its effects by depolymerizing microtubules. Herein we report a structural analog of nocodazole, a (1*H*-pyrrol-1-yl)methyl-1*H*-benzoimidazole carbamate ester derivative, named RDS 60. We evaluated the antineoplastic properties of RDS 60 in two human head and neck squamous cell carcinoma (HNSCC) cell lines and we found that this compound significantly inhibited replication of both HNSCC cell lines without inducing any important cytotoxic effect on human dermal fibroblasts and human keratinocytes. The treatment of HNSCC cell lines with 1 μM RDS 60 for 24 h stopped development of normal bipolar mitotic spindles and, at the same time, blocked the cell cycle in G2/M phase together with cytoplasmic accumulation of cyclin B1. Consequently, treatment with 2 μM RDS 60 for 24 h induced the activation of apoptosis in both HNSCC cell lines. Additionally, RDS 60 was able to reverse the epithelial-mesenchymal transition and to inhibit cell migration and extracellular matrix infiltration of both HNSCC cell lines. The reported results demonstrate that this compound has a potent effect in blocking cell cycle, inducing apoptosis and inhibiting cell motility and stromal invasion of HNSCC cell lines. Therefore, the ability of RDS 60 to attenuate the malignancy of tumor cells suggests its potential role as an interesting and powerful tool for new approaches in treating HNSCC.

## 1. Introduction

Nocodazole is a benzimidazol-2-yl carbamate ester derivative that interacts with microtubules by targeting tubulin. The high degree of affinity of nocodazole for beta tubulin induces the development of hydrogen bonds between the single beta subunits, interfering with the physiologic aggregation of alpha and beta subunits into the tubulin heterodimer [1,2]. The failure to form tubulin heterodimers induces the depolymerization of microtubules, determining an alteration of mitotic spindle organization with the incapacity to carry out mitosis [3,4]. Whenever high replication rate cells are treated with nocodazole, they are not able to organize a normal bipolar mitotic spindle and do not complete mitosis, remaining blocked in the G2 or M phase of the cell cycle. Furthermore, the prolonged arrest in pro-metaphase is a starting point for apoptosis [5]. The high specificity to activate apoptotic death exclusively for high replication rate cells makes nocodazole an interesting anticancer molecule. For example, nocodazole induces apoptosis by microtubule depolymerization in leukemic cells from chronic lymphocytic leukemia patients, without affecting the viability of T-lymphocytes and mesenchymal stromal cells derived from the same patients [6]. A pre-clinical in vivo study has shown a significant therapeutic anticancer efficacy of nocodazole, also potentiated by ketoconazole, in human colon carcinoma xenografts in nude mice [7]. However, despite its antitumor activity, nocodazole has not yet reached the clinical phase [8]. One of the reasons could be that nocodazole induces apoptosis of erythrocytes, although at concentrations much higher than those required for disassembly of microtubules, and this can cause toxicity in clinical conditions [9].

**Figure 1:**
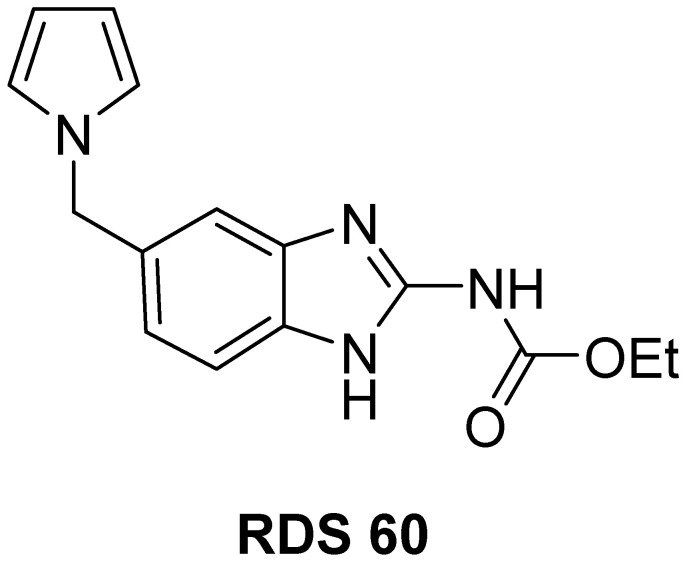
Chemical structure of RDS 60.

On the other hand, several reports have suggested that benzimidazole is the most prominent heterocycle, having good cytotoxic properties against different types of cancer [10,11,12]. Actually, many different benzimidazole-based drugs acting as colchicine binding site inhibitors of tubulin have been applied not only in pre-clinical, but also in clinical, studies, and have revealed interesting activity for cancer treatment [13]. In addition, nocodazole targets to the colchicine binding site of beta tubulin subunit, impairing its assembly with alpha tubulin subunits. However, unlike colchicine, the binding of nocodazole to tubulin is rapidly reversible [14]. Therefore, we decided to deepen our knowledge on the anti-tumoral activity of a (1*H*-pyrrol-1-yl)methyl-1*H*-benzoimidazole derivative structurally related with nocodazole that we previously synthesized and reported [15], namely RDS 60 (Figure 1).

To this aim, we evaluated the efficiency of RDS 60 as an anti-tumoral agent towards human head and neck squamous cell carcinoma (HNSCC), a group of tumors detectable in various regions of the oral cavity, oropharynx, hypopharynx and larynx. We chose this type of neoplasm because it is extremely aggressive; in fact, almost 50% of newly diagnosed HNSCC patients have a survival rate below 5 years [16]. HNSCC are collocated at the sixth position among more diffuse malignant tumors in the world [17], being characterized by a poor prognosis, mainly due to metastases, the development of multiple primary tumors, and local and regional, often inoperable, relapses [18,19]. The presence of distant metastases at the moment of diagnosis is associated with a high mortality rate for HNSCC [20], since the chemotherapy used for metastatic disease gives often low responses [21,22].

Herein, we demonstrated that two HNSCC cell lines displayed an important dose-and time-dependent reduction in cell viability when treated with our benzimidazol-2-yl carbamate ester RDS 60. In addition, this novel compound was capable of impairing tubulin assembly into the mitotic spindle, blocking the cell cycle in G2 phase, inducing apoptosis, and reversing the invasive phenotype of both HNSCC cell lines.

## 2. Results

### 2.1. Chemistry

**Scheme 1:**
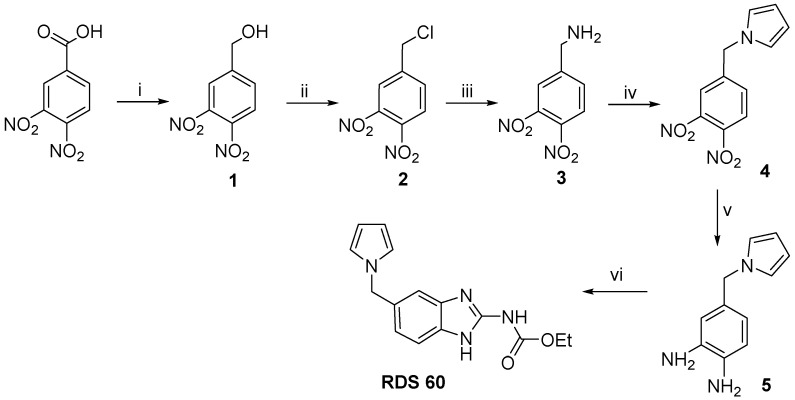
Synthetic route to RDS 60. i: AlCl_3_, NaBH_4_, THF dry, 0 °C to reflux, 3 h and 20 min; ii: PCl_5_, CHCl_3_, 0 °C to RT, 2 h and 45 min; iii: 1. (1s,3s)-1,3,5,7-tetraazaadamantane, NaI, EtOH abs., CHCl_3_, RT, 24 h, 2. HCl (37%), EtOH, 55 °C, 4 h, 3. Na_2_CO_3(ss)_, RT; iv: 2,5-dimethoxytetrahydrofuran, AcOH, reflux, 5 min; v: H_2_/Pd, AcOEt, 45 °C, 4 h; vi: S-methylisothiourea, PTSA, EtOH, reflux, 45 min.

Given the antitumoral activity of the benzimidazole-based small molecule nocodazole, we decided to deepen our understanding of the activity of RDS 60, a nocodazole analog belonging to our in-house library. Indeed, this compound shares the same benzimidazol-2-yl carbamate ester structure of nocodazole and differs in the replacement of the 2-thienyl substituent with its isostere 1-pyrrolyl ring and in the reduction of the carbonyl group with a methylene one (Figure 1). RDS 60 (ethyl (5-((1*H*-pyrrol-1-yl) methyl)-1*H*-benzo(*d*)imidazol-2-yl)carbamate) was synthesized as previously described [15]. However, we modified the first two reaction steps in order to improve the overall reaction yields. Indeed, we applied a reduction reaction according to Moore et al. [23] of the commercially available carboxylic acid into alcohol (compound **1**) that was subsequently converted in the corresponding benzyl chloride (**2**) via an aliphatic nucleophilic substitution (Scheme 1). The subsequent synthetic steps resemble the ones previously reported [15]. Therefore, we report the details of the first two synthetic steps since the other ones can be found in ref. [15].

### 2.2. RDS 60 Inhibits Proliferation and Impairs Mitotic Spindle Formation in HNSCC Cell Lines

**Figure 2:**
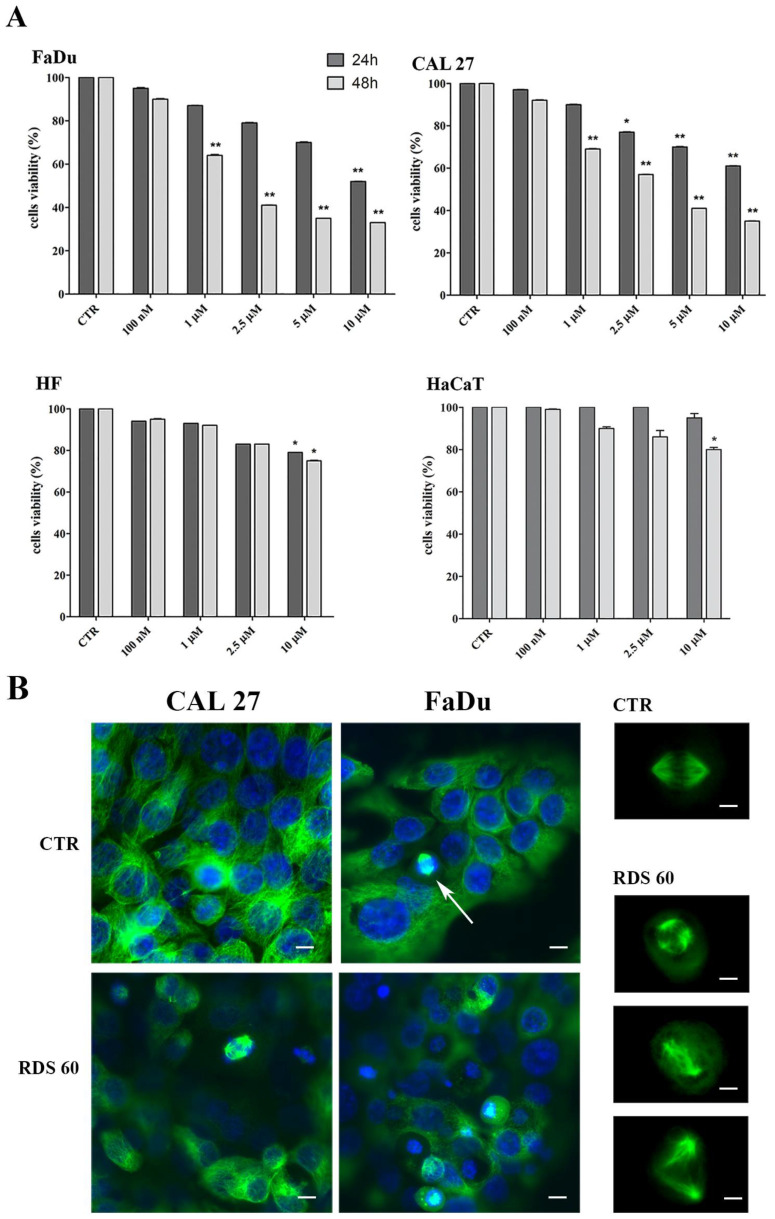
Effects of RDS 60 on proliferation and mitotic spindle formation. (**A**) Cell viability of untreated (CTR) and RDS 60 treated FaDu, CAL27, HF and HaCaT expressed as percentages of alive cells ± SD. Treatments were performed for 24 and 48 h. * *p* < 0.01; ** *p* < 0.001. (**B**) Immunofluorescence staining of beta tubulin on CAL27 and FaDu untreated (CTR) and 1 μM RDS 60 treated for 24 h; bar 5 μm. The arrow shows a normal mitotic spindle. Smaller panels show mitotic spindles at higher magnification from untreated (CTR) and RDS 60 treated cells; bar 2 μm.

To evaluate the effects of this new compound on tumor cell viability and to investigate its possible cytotoxicity on normal somatic cells, two human tumor cell lines, tongue HNSCC CAL 27 and pharynx HNSCC FaDu, and two human non-transformed cell lines, dermal fibroblasts HF and keratinocytes HaCaT, were chosen. The two HNSCC cell lines were treated for 24 and 48 h with 100 nM, 1 μM, 2.5 μM, 5 μM, and 10 μM RDS 60. A significant decrease in cell viability was seen at 24 h of RDS 60 treatment when used at 2.5 μM for CAL 27 and at 10 μM for FaDu; the decrease of proliferation became significant at 1 μM RDS 60 for both tumor cell lines at 48 h of treatment (Figure 2A). We repeated the time points of RDS 60 concentrations at 1, 10, 20, 30, and 40 μM to calculate the EC_50_ that resulted; 10.8 μM for CAL27 and 12.4 μM for FaDu at 24 h; 2.5 μM for CAL27 and 2.9 μM for FaDu at 48 h. To evaluate the cytotoxicity of the compound on normal somatic cells, human dermal fibroblast HF and human keratinocyte HaCaT cell lines were treated for 24 and 48 h with 100 nM, 1 μM, 2.5 μM and 10 μM RDS 60. RDS 60 treatment led to decreased viability of HF and HaCaT only at the highest concentration of 10 μM (Figure 2A). Therefore, we decided to perform the following experiments on tumor cells utilizing the compound at low concentrations, between 1 and 2 μM, corresponding to non-cytotoxic doses for HF and HaCaT.

Nocodazole, analog of RDS 60, determines the impairment of regular organization of microtubules by targeting tubulin. Therefore, we analyzed whether RDS 60 could interfere with tubulin assembly into the mitotic spindle. To understand this aspect, we performed an immunofluorescent staining of beta-tubulin in both HNSCC cell lines untreated and treated with 1 μM RDS 60 for 24 h and we observed the cells undergoing mitosis. We found that all untreated cells developed normal typical bipolar mitotic spindles, while it was impossible to find normal mitotic spindles in the cells treated with RDS 60, because every mitotic spindle had abnormal shape and size, with tripolar or multipolar structure, certainly determining abortive mitosis (Figure 2B). In order to quantify the mitotic index, the mitotic spindles were counted and related to the total number of cells per field. The mitotic index was 5% for basal CAL27 and 4% for treated CAL27, and 7% for basal FaDu and 5% for treated FaDu. As expected, the mitotic index did not change significantly upon RDS 60 treatment, but every mitosis was irregular and abortive in treated cells. We also performed tubulin immunofluorescence on HF and observed that all mitotic spindles appeared normal either in untreated or in 1 μM RDS 60 treated fibroblasts (data not shown).

### 2.3. RDS 60 Determines a Block in G2/M and Up-Regulates Cytoplasmic Cyclin B1 in HNSCC Cell Lines

**Figure 3:**
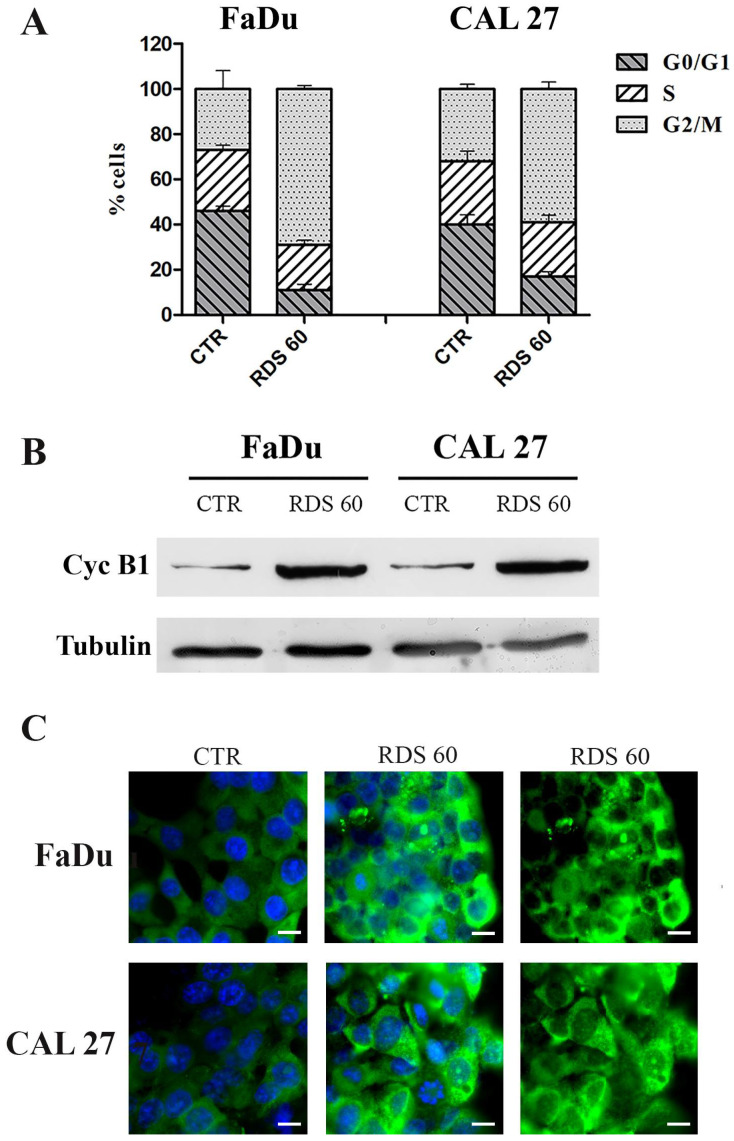
Block in G2/M and up-regulation of cytoplasmic cyclin B1 in HNSCC cell lines induced by RDS 60. (**A**) Cell cycle evaluated by flow cytometry of FaDu and CAL27 untreated (CTR) and 1 μM RDS 60 treated for 24 h. The histogram shows the media ± SD representative of results obtained from three independent experiments. (**B**) Western blot of cyclin B1 from FaDu and CAL27 untreated (CTR) and 1 μM RDS 60 treated for 24 h. (**C**) Immunofluorescence staining of cyclin B1 on FaDu and CAL27 untreated (CTR) and 1 μM RDS 60 treated for 24 h shown with and without nuclear staining; bar 7 μm.

We then investigated whether RDS 60 could hamper any cell cycle checkpoint, so we analyzed both HNSCC cell lines by flow cytometry and found that treatment with 1 μM RDS 60 for 24 h induced a notable increase of cells in the G2/M phase and a parallel decrease of cells in the S and G0/G1 phases. Specifically, RDS 60 increased the percentage of cells in the G2/M phase from 27% in untreated to 69% in treated FaDu and from 32% to 56% respectively in untreated and treated CAL27 (Figure 3A). In parallel, we evaluated cyclin B1 expression, since an increase of this protein is essential for the regular control of cell cycle transition from S to G2 and then to the M phase [24]. We found that cyclin B1 was up-regulated in both HNSCC cell lines upon 1 μM RDS 60 treatment for 24 h, as evidenced by western blot analysis (Figure 3B). We also observed cyclin B1 by immunofluorescence and found that its staining was significantly enhanced after 1 μM RDS 60 treatment for 24 h and that its localization was exclusively cytoplasmic (Figure 3C). The fact that cyclin B1 increased in the cytoplasm without translocating into the nucleus was in accordance with the increase of cells blocked in the G2/M phase.

### 2.4. RDS 60 Induces Apoptosis in HNSCC Cell Lines

**Figure 4:**
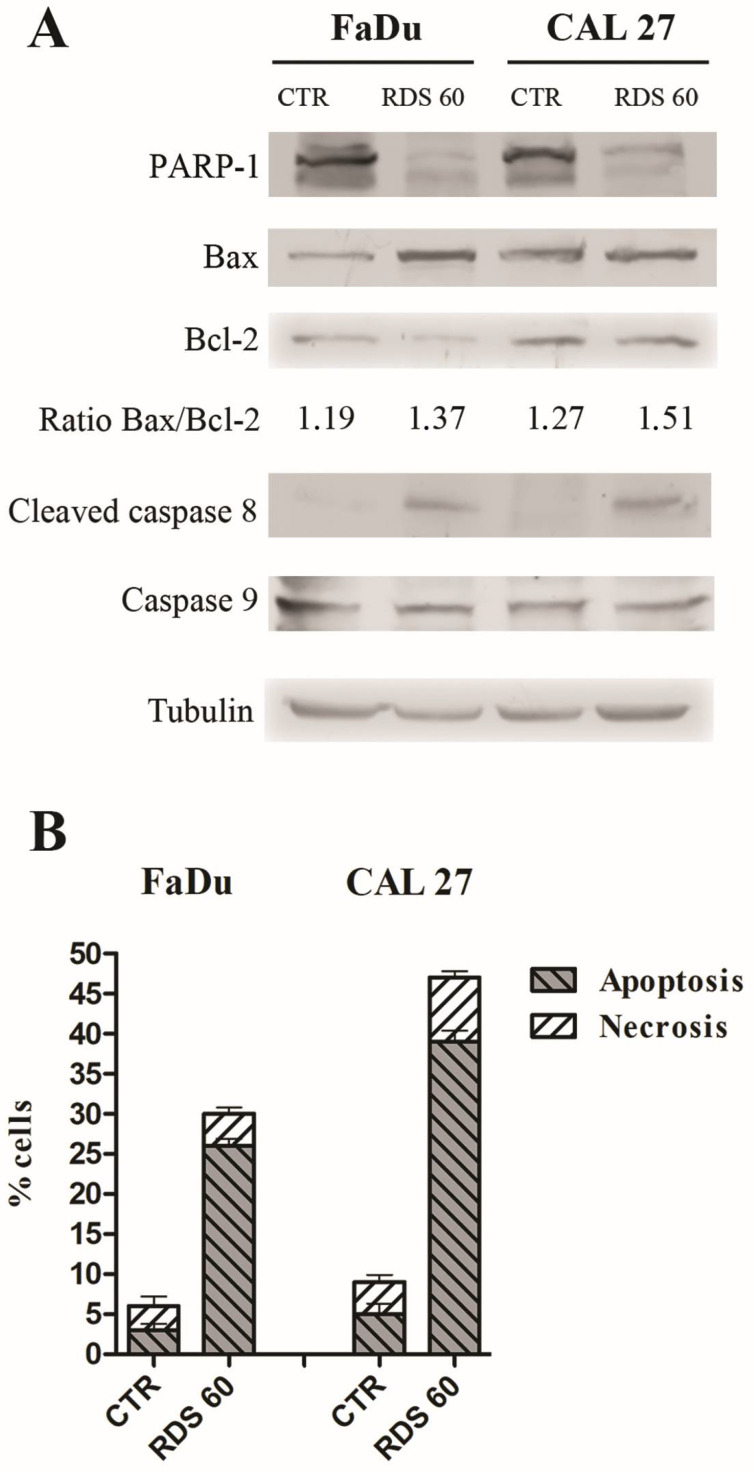
Apoptosis induced by RDS 60 in HNSCC cell lines. (**A**) Western blot of PARP-1, Bax, Bcl-2, cleaved caspase 8 and caspase 9 from FaDu and CAL27 untreated (CTR) and 2 μM RDS 60 treated for 24 h. The means from densitometry quantifications of three different experiments of the Bax to Bcl-2 ratio normalized to tubulin are indicated. (**B**) Percentages of apoptotic and necrotic cells evaluated by annexin V-APC and 7AAD expression quantified by FACS on FaDu and CAL27 untreated (CTR) and 2 μM RDS 60 treated for 24 h. The histogram shows the media ± SD representative of results obtained from two independent experiments.

Since the block of cell cycle can represent a starting point for apoptosis, we evaluated whether RDS 60 could induce apoptosis. Therefore, the expression of PARP-1 was analyzed, because its cleavage by caspase 3 occurs during the late phases and the diminution of full-length PARP-1 is considered a marker of apoptosis. An important decrease in PARP-1 was observed in both HNSCC when treated with 2 μM RDS 60 for 24 h (Figure 4A). In addition, two other proteins were evaluated: Bcl-2, an anti-apoptotic protein, and Bax, a pro-apoptotic Bcl-2-associated protein. We found that the pro-apoptotic protein Bax was up-regulated in both cell lines treated with 2 μM RDS 60 for 24 h, while the anti-apoptotic protein Bcl-2 was down-regulated in treated FaDu. The densitometic values of Bax and Bcl2 bands were then measured and the Bax/Bcl2 ratio was calculated and shown to be increased in both HNSCC cell lines upon RDS 60 treatment (Figure 4A). This data confirmed the apoptotic response, because an increased Bax/Bcl-2 ratio is indicative of apoptosis. Lastly, the activation of initiator caspases 8 and 9 was analyzed, since the presence of proteolytic fragments of caspases is indicative of their activation and is an additional marker of apoptosis. Cleaved caspase 8 appeared in both cell lines after RDS 60 treatment, while caspase 9 was not modified by the treatment (Figure 4A), suggesting that RDS 60-induced apoptosis follows the extrinsic activation pathway mediated by caspase 8.

To quantify tumor cell death induced by RDS 60, apoptosis and necrosis rates were evaluated. Therefore, both HNSCC cell lines untreated and treated with 2 μM RDS 60 for 24 h were double stained with APC conjugated annexin V and 7AAD and analyzed by flow cytometry. Annexin V staining, indicative of apoptosis, increased significantly after RDS 60 treatment, from 3% to 26% in FaDu and from 5% to 39% in CAL27 (Figure 4B), while the necrosis rate did not modify significantly.

### 2.5. RDS 60 Reverses EMT and Inhibits Migration of HNSCC Cell Lines

**Figure 5:**
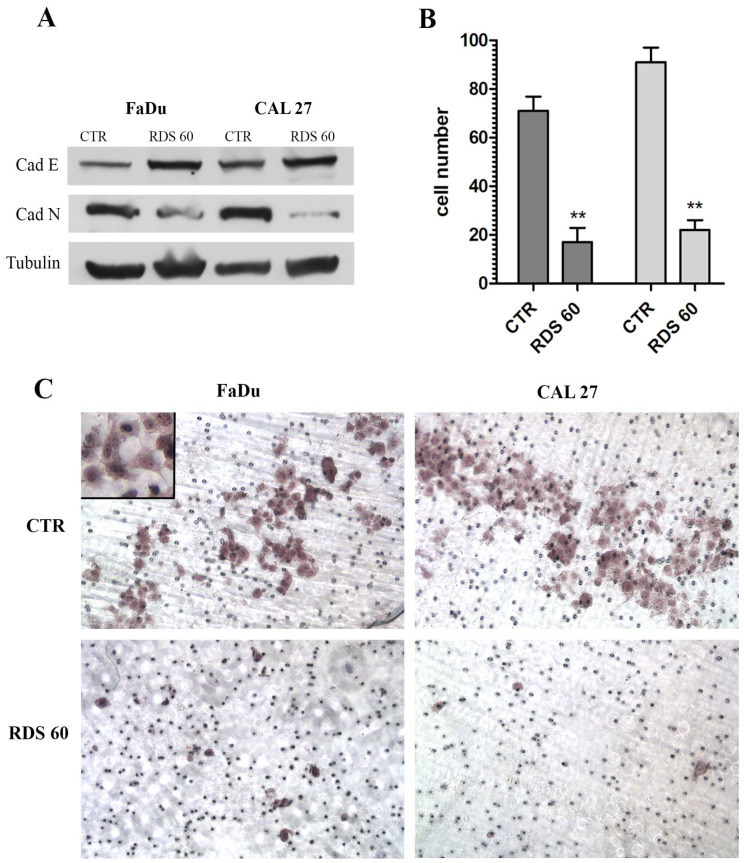
EMT reversion and migration inhibition of HNSCC cell lines induced by RDS 60. (**A**) Western blot of EMT related proteins E-cadherin and N-cadherin from untreated (CTR) and 1 μM RDS 60 treated for 24 h FaDu and CAL27. (**B**) Graphic representing the number of migrated cells in five random fields from three independent experiments of Matrigel invasion assay with the means ± SD; ** *p* < 0.001. (**C**) Matrigel invasion assay of untreated FaDu and CAL27 cells (CTR) and 1 μM RDS 60 treated cells for 16 h. The higher magnification insert on the top left angle shows morphological heterogeneity and mesenchymal phenotype of untreated FaDu.

We then investigated the epithelial-mesenchymal transition (EMT), which is a fundamental feature of malignant cells. Therefore, the modulation of two important markers of EMT, E-cadherin and N-cadherin, was analyzed by Western blot. E-cadherin was low under basal conditions and increased after 1 μM RDS 60 treatment for 24 h in both HNSCC cell lines, and at the same time, N-cadherin was highly expressed in both untreated HNSCC and almost disappeared after treatment (Figure 5A). This result showed that RDS 60 was able to reverse the EMT phenotype in both HNCSS cell lines.

In order to quantify the degree of malignancy of these tumor cells, a Matrigel invasion assay was performed. The number of cells able to migrate through the Matrigel were counted and shown to dramatically diminish after 1 μM RDS 60 treatment for 16 h. In detail, migrating FaDu cells decreased from 71 to 17 and migrating CAL27 from 92 to 23 (Figure 5B). This result demonstrated that RDS 60 determined an important inhibition of HNSCC invasion capacity, decreasing the number of cells capable of crossing through the Matrigel by 76% for FaDu and 75% for CAL27. This was confirmed by the microscopy images of the Matrigel invasion assay, where both untreated HNSCC cell lines showed a high density of migrated cells, while 1 μM RDS 60 treatment for 16 h significantly diminished the number of cells capable of migrating (Figure 5C). The heterogeneous morphology and the mesenchymal phenotype, hallmarks of EMT, were also visible in untreated FaDu (Figure 5C).

Taken together, these data demonstrated that RDS 60 played an important role not only in blocking the cell cycle and entering apoptosis, but also in reducing the invasive phenotype of HNSCC cells.

## 3. Discussion

Over the last decades, microtubules have constituted an interesting target in cancer therapy and the research is still ongoing to develop new microtubule-targeting agents in order to increase their selectivity for tumor cells and decrease their unwanted side effects [25,26].

Given our interest in developing new anti-cancer agents, we chose to design and synthesize a derivative of nocodazole, a microtubule targeting compound, which affects cycling cells causing mitotic arrest and apoptosis. We evaluated the anti-tumoral activity of this compound, named RDS 60 on two HNSCC cell lines and we demonstrated that it was a potent inhibitor of proliferation for both HNSCC cell lines, while it did not exert any important cytotoxic effects on human dermal fibroblasts and keratinocytes. The higher selectivity of RDS 60 toward tumor cells may be due to a specific activity on rapidly cycling cells, rather than slow cycling cells. The described selective cytotoxicity of RDS 60 to HNSCC cell lines, but not to nonmalignant fibroblasts and keratinocytes, is an interesting feature for possible therapeutic application.

We illustrated that RDS 60 induced a negative regulation of tumor cell growth by targeting beta tubulin and hampering the assembly of bipolar mitotic spindles with the following abortion of mitosis. The microtubule depolymerizing drug nocodazole inhibits proliferation through a block of cell cycle in G2/M [27]. In a similar way, RDS 60 elicited G2/M phase cell cycle arrest and contemporaneously an impressive accumulation of cyclin B1 into the cytoplasm. These two events are in accordance, because it is well known that cyclin B1 increases when the cell is approaching G2 phase; following this, it is phosphorylated and translocates from the cytoplasm to the nucleus before M phase, in order to initiate mitosis [24]. We observed that cyclin B1 increased upon RDS 60 treatment, but it remained totally localized in the cytoplasm. This information suggested that cyclin B1 was not activated and that, for this reason, the cell cycle was blocked in G2 phase, in particular it was interrupted between G2 and M phase.

Usually, a cell undergoing a prolonged arrest in pro-metaphase starts towards apoptosis [5]; likewise, RDS 60 acted as apoptosis inducer for both HNSCC cell lines, as demonstrated by the cleavage of PARP-1 and by the increase of the Bax/Bcl-2 ratio. RDS 60 dependent apoptosis followed the extrinsic pathway, as demonstrated by the appearance of cleaved caspase 8 and by the contemporaneous lack of cleavage of caspase 9.

We then studied some features of HNSCC malignant and aggressive phenotype and found that both cell lines basally expressed low rates of E-cadherin and high rates of N-cadherin. In cancer, the loss of E-cadherin and the appearance of N-cadherin are considered among the key steps in EMT. This transition toward a mesenchymal-like phenotype gives migratory and invasive ability to tumor cells [28]. We demonstrated that RDS 60 was able to reverse this EMT behavior, by up-regulating E-cadherin and down-regulating N-cadherin in both HNSCC cell lines. EMT is an important step in tumor progression, because it results in cells capable of motility and infiltration, which are all characteristics reflecting the clinical aggressive behavior of these tumors [29,30]. In fact, quantifying the in vitro invasion potential, both HNSCC cell lines showed a high basal rate of infiltration into the extracellular matrix performed with the Matrigel invasion assay. As expected, the treatment with RDS 60 markedly impaired cell motility and infiltration, reducing the invasion capacity up to four fold in both HNSCC cell lines.

RDS 60 ability to reduce invasiveness can be related to its specific target represented by tubulin, which is the constituent not only of stable microtubules, but also of dynamic ones; consequently, any kind of tubulin alteration can reduce microtubule stability [31]. For example, it has been shown that post-translational deacetylation of tubulin alters the microtubule structure and promotes cell motility; in fact, the deacetylation of tubulin obtained with the overexpression of histone deacetylase 6 (HDAC6) is sufficient to enhance fibroblast motility [32]. We can hypothesize that RDS 60, through the destabilization of tubulin, can interfere with dynamic microtubule organization and consequently decrease HNSCC cell mobility.

Taken together these data suggest that RDS 60 could represent a potential innovative anticancer agent for HNSCC, since this molecule is able to reduce tumor cell viability by blocking cells in the G2 phase, induce apoptosis and direct the invasive cells toward a less malignant phenotype. The effectiveness of other better-known microtubule-targeting agents in the clinical setting of cancer therapy can be reduced by different side effects, for example hematological and neurological toxicities and also by the development of drug resistance [33]. Therefore, when the next step in the study of this new molecule will be performed on animal models, it could be that some unwanted side effects could occur.

In conclusion, we can present RDS 60 as a valuable additional tool to control and attenuate HNSCC malignity and aggressiveness.

## 4. Materials and Methods

### 4.1. Chemistry

#### 4.1.1. General Instrumentation

Melting points were determined on a Bobby Stuart Scientific SMP1 melting point apparatus (Bibby Scientific, Stone, UK) and are uncorrected. Compound purity was always >95% as determined by combustion analysis. Analytical results agreed to within ±0.40% of the theoretical values. IR spectra were recorded on a PerkinElmer Spectrum-One spectrophotometer (Perkin Elmer, Shelton, CT, USA). ^1^H NMR spectra were recorded at 400 MHz on a Bruker AC 400 Ultrashield 10 spectrophotometer (400 MHz) (Bruker, Billerica, MA, USA). Dimethyl sulfoxide-*d*_6_ 99.9% (CAS 2206-27-1) and deuterochloroform 98.8% (CAS 865-49-6) of isotopic purity (Aldrich, St. Louis, MO, USA) were used. Column chromatographies were performed on silica gel (Merck, Darmstadt, Germany; 70−230 mesh) and on aluminum oxide (Merck; 70–230 mesh). All compounds were routinely checked on TLC by using aluminum-baked silica gel plates (Fluka, Honeywell, Charlotte, NC, USA; DC-Alufolien Kieselgel 60 F_254_) or TLC aluminum oxide 60 F_254_ basic (Merck). Developed plates were visualized by UV light. Solvents were reagent grade and, when necessary, were purified and dried by standard methods. Concentration of solutions after reactions and extractions involved the use of a rotary evaporator (Büchi, Flawil, Switzerland) operating at a reduced pressure (ca. 20 Torr). Organic solutions were dried over anhydrous sodium sulfate (Merck). All solvents were freshly distilled under nitrogen and stored over molecular sieves for at least 3 h prior to use. Melting point (°C), recrystallization solvent, yield (%), chromatographic system, IR, ^1^H NMR, formula, Mr and analyzed elements for intermediates **1**–**5** and derivative RDS 60 agreed with the ones previously described [15]. RDS 60 samples used for biological evaluation were 99% pure as determined by elemental analysis.

#### 4.1.2. Specific Procedures and Characterization

(3,4-Dinitrophenyl)methanol (**1**). A solution of AlCl_3_ (3.77 g; 28.28 mM) in THF dry (4.6 mL) was added dropwise into a solution of commercially available 3,4-dinitrobenzoic acid (5 g; 23.57 mM) and NaBH_4_ (3.21 g; 84.85 mM) in THF dry (46 mL) at 0 °C within 20 min. The reaction was stirred at 25 °C for 1 h (h) and for 2 h to reflux and then poured into ice-cold water (115 mL). The pH was adjusted to 7 with 1 N HCl and the mixture was extracted with ethyl acetate (3 × 50 mL). The collected extracts were washed with brine (3 × 100 mL) and dried. Removal of the solvent furnished crude residue that was washed with ethyl acetate in order to discard the boron salt, yielding intermediate 1 (3.37 g; 72.1%) as a yellow solid.

4-(Chloromethyl)-1,2-dinitrobenzene (**2**). To a solution of compound **1** (16.27 g; 81.85 mM) in CHCl_3_ (126 mL) was added PCl_5_ (6.63 g; 31.85 mM) portion-wise at 0 °C within 15 min. The reaction mixture was stirred at 0 °C for 15 min and at 25 °C for 2.5 h and then poured into ice-cold water. The organic layer was washed with 5% *w*/*v* Na_2_CO_3_ (3 × 100 mL) and with NaCl_ss_ (3 × 100 mL) and dried. Removal of the solvent furnished the crude compound **2** that underwent the following step without further purification.

(3,4-Dinitrophenyl)methanamine (**3**). Compound **3** was prepared according to the literature [10]. Analytical data are herein reported.

1-(3,4-Dinitrobenzyl)-1*H*-pyrrole (**4**). Compound **4** was prepared according to the literature [10]. Analytical data are herein reported.

(4-((1*H*-pyrrol-1-yl)methyl)benzene-1,2-diamine (**5**). Compound **5** was prepared according to the literature [10]. Analytical data are herein reported.

Ethyl (5-((1*H*-pyrrol-1-yl)methyl)-1*H*-benzo[*d*]imidazol-2-yl)carbamate (RDS 60). RDS 60 was prepared according to the literature [15]. Analytical data are herein reported.

### 4.2. Cell Cultures and Treatment

The following cell lines were utilized: tongue HNSCC CAL27 (CRL-2095, ATCC, Manassas, VA, USA), pharynx HNSCC FaDu (HTB-43, ATCC, USA), human keratinocytes HaCaT (ATCC, USA) and human dermal fibroblasts HF previously established and characterized in our laboratory [34]. Mycoplasma testing was done for all cell lines at the beginning of the study. Cells were grown in RPMI 1640 medium with 10% fetal calf serum (FCS), 2 mM glutamine and 50 U/mL penicillin-streptomycin (Sigma-Aldrich). The compound RDS 60 was solubilized in dimethylsulfoxide (DMSO) (Sigma) for a 10 mM stock solution and utilized to final concentrations from 100 nM to 10 μM for 24 and 48 h. Control cells were treated with equivalent amounts of DMSO in every experiment.

### 4.3. Cytotoxicity Assay

To determine cytotoxicity, a sulforhodamine-B colorimetric assay was performed: 5 × 10^3^ cells were plated in 96-well plates, grown for 24 h and then treated with 100 nM, 1, 2.5, 5, 10 μM RDS 60 for 24 and 48 h. Cells were then fixed with 50% trichloroacetic acid for 1 h at 4 °C and stained for 30 min at room temperature (RT) with 0.4% sulforhodamine-B in 1% acetic acid. Excess dye was removed by washing four times with 1% acetic acid. Protein-bound dye was dissolved in 10 mM Tris pH 10 and optical density was determined at 510 nm using a microplate reader.

### 4.4. Flow Cytometry Analysis

Cells were grown on 60-mm plates for 24 h and then treated with 1 μM (for cell cycle analysis) or 2 μM (for apoptosis analysis) RDS 60 or equivalent amounts of DMSO for 24 h. Detached and adherent cells were harvested by trypsinization and washed twice with cold PBS. For apoptosis and necrosis rate quantification, the cells were double stained with Annexin V-APC (allophycocyanin) and 7AAD (7-amino-actinomycin) in Ringer solution containing 5 mM CaCl_2_ for 1 h at 4 °C according to the manufacturer’s instructions (BD Biosciences, Apoptosis kit, Franklin Lakes, NJ, USA) and analyzed by a fluorescence activated cell sorting cytofluorimeter (FACS) FACSCalibur (BD Biosciences) and by the Cell-Quest Pro software version 5.1 (BD Biosciences). For cell cycle analysis, the cells were fixed in 70% ethanol overnight at 4 °C and then were rinsed twice with PBS and incubated with 50 µL of RNAse (100 µg/mL, Sigma, Kawasaki, Japan) to ensure that only DNA was stained, and 200 µL of propidium iodide (PI) (50 µg/mL, Sigma). Cell staining analysis was performed using the FACS CantoII equipped with 488 nm laser and DIVA Software (BD Biosciences). The cells were first gated using a forward vs. side scatter (FSC vs. SSC) strategy and upon 488 nm laser excitation, then Annexin V-APC and PI fluorescence was detected above 580 nm. Data were analyzed using Flow Jo software (Flow Jo LLC, Ashland, OR, USA).

### 4.5. Immunofluorescence

Cells were grown on Nunc Labteck chamber slides for 24 h and then treated with 1 μM RDS 60 or DMSO for 24 h. Cells were washed with PBS with Ca/Mg (washing buffer) and fixed with 4% buffered paraformaldehyde (Sigma Aldrich) for 20 min at 4 °C, then permeabilized with PBS, 5% FCS, 0.5% TritonX100 for 30 min at RT and incubated for 1 h to RT with the primary rabbit polyclonal antibody to beta-tubulin (1:200 diluted; Immunological Sciences, Rome, Italy). Alternatively, cells were permeabilized with 0.1% TritonX100 for 10 min to RT, incubated with 3% BSA for 1 h at RT and incubated with the primary rabbit polyclonal antibody to cyclin B1 in 0.1% BSA (1:200 diluted; Elabscience, Houston, TX, USA) overnight at 4 °C. Cells were washed twice with washing buffer and incubated with the secondary anti-rabbit antibody FITC conjugated (1:400 diluted; Molecular Probes, Eugene, OR, USA) for 1 h at RT. Cells were washed twice with washing buffer and DNA was stained with Hoechst for 15 min at RT. The slide was mounted with ProLong-Antifade (Life Technologies, Carlsbad, CA, USA) and analyzed by a fluorescence microscope (Olympus BX52, Olympus America, Inc., Melville, NY, USA). Image acquisition and processing were conducted by IAS 2000 software.

### 4.6. Western Blot Analysis

Cells were grown in 100-mm plates for 24 h and treated with 2 μM RDS 60 or equivalent amounts of DMSO for 24 h. Cells were then scraped in lysis buffer composed of 1% Triton, 0.1% sodium dodecyl sulfate, 150 mM NaCl, 50 mM Tris HCl pH 7.4, 2 mM EDTA with protease inhibitor cocktail (Roche Applied Science, Penzberg, Germany) for 30 min at 4 °C. Lysates were centrifuged at 16,000× *g* for 15 min at 4 °C and the supernatant was collected. Protein concentration was evaluated using Protein Concentration Assay (Bio-Rad Laboratories, Hercules, CA, USA). Protein lysates (50–100 μg) were separated by molecular weight with 10, 12 or 14% SDS-PAGE and then transferred onto nitrocellulose membranes. Membranes were blocked for 1 h at RT in 5% nonfat dry milk and incubated with primary antibodies opportunely diluted in PBS-Tween overnight at 4 °C, then washed in Tris-buffered saline with 0.1% Tween-20 and incubated with horseradish-peroxidase-conjugated-anti-mouse/rabbit-IgG (1:5000; Sigma-Aldrich, St. Louis, MO, USA) for 1 h at RT. Filters were finally developed using enhanced chemiluminescence (Super Signal West Pico Chemiluminescence Substrate; Thermo Fisher Scientific, Waltham, MA, USA) using Kodak X-Omat films (Kodak, Rochester, NY, USA). Primary antibodies were: mouse anti-poly (ADP-ribose)-polymerase (PARP-1) (diluted 1:500; Santa Cruz Biotechnology, Dallas, TX, USA); mouse anti-cleaved-caspase 8 (diluted 1:500; Cell Signaling Technology, Danvers, MA, USA); mouse anti-caspase 9 (diluted 1:500; Cell Signaling Technology); mouse anti-B-cell-lymphoma-2 (Bcl-2) (diluted 1:200; Santa Cruz Biotechnology); rabbit anti-Bcl-2-associated-X-protein (Bax) (diluted 1:250; Santa Cruz Biotechnology); rabbit anti E-cadherin (1:1000 diluted; GeneTex, Irvine, CA, USA); rabbit anti N-cadherin (1:1000 diluted; GeneTex); rabbit anti cyclin B1 (1:500 diluted; Elabscience, USA); rabbit anti-tubulin (diluted 1:4000; Immunological Sciences). Experiments were performed in triplicate, the bands from the blots were quantified using ImageJ v.1.48 software (National Institutes of Health, Bethesda, MD, USA), the mean values were calculated and expressed as densitometric units (DU).

### 4.7. Invasion Assay

Invasion assay was performed with Matrigel Invasion Chambers (Corning, Corning, NY, USA) consisting of inserts with 8 μm pore membranes pretreated with Matrigel. Cells at a density of 5 × 10^5^/mL were plated in serum-free medium plus DMSO or in serum-free medium plus 1 μM RDS 60 in the insert chamber, the lower chamber contained complete medium with FCS. After 18 h of culture the inserts were washed with PBS with Ca/Mg and fixed with 100% methanol for 20 min at 4 °C, washed twice with PBS with Ca/Mg and stained for 20 min at RT with hematoxylin. The inserts were then mounted on slides and the cells that migrated through the filter pores to the lower side of the membrane were counted by an optical microscope (Olympus BX52). Image acquisition and processing were conducted by IAS 2000 software.

### 4.8. Statistical Analysis and Graphic Programs

All results were analyzed using one-way analysis of variance, and significance was evaluated using Tukey’s honest significant difference post hoc test. All figures were created using Adobe Photoshop CS5 and all graphs were produced and statistical analyses conducted using Graph Pad Prism 5.0.

## Data Availability

The data presented in this study are available on request from the corresponding author.

## Sample Availability

Samples of the compounds **1**–**5** and RDS 60 are available from the authors.
